# The Associations between Insomnia Severity and Health Outcomes in the United States

**DOI:** 10.3390/jcm12062438

**Published:** 2023-03-22

**Authors:** François-Xavier Chalet, Paul Saskin, Ajay Ahuja, Jeffrey Thompson, Abisola Olopoenia, Kushal Modi, Charles M. Morin, Emerson M. Wickwire

**Affiliations:** 1 Idorsia Pharmaceuticals Ltd., Hegenheimermattweg 91, 4123 Allschwil, Switzerland; 2 Idorsia Pharmaceuticals US Inc., One Radnor Corporate Center, Suite 101, 100 Matsonford Rd, Radnor, PA 19087, USA; 3 Cerner Enviza, 51 Valley Stream Pkwy, Malvern, PA 19355, USA; 4 Department of Psychology, Cervo Brain Research Centre, Laval University, Québec City, QC G1V 0A6, Canada; cmorin@psy.ulaval.ca; 5 Sleep Disorders Center, Division of Pulmonary and Critical Care Medicine, Department of Medicine, University of Maryland School of Medicine, Baltimore, MD 21201, USA; ewickwire@som.umaryland.edu; 6 Department of Psychiatry, University of Maryland School of Medicine, Baltimore, MD 21201, USA

**Keywords:** insomnia severity, health-related quality of life, health utility status, workplace productivity, healthcare resource utilization, NHWS

## Abstract

Little is known about the associations between insomnia severity, insomnia symptoms, and key health outcomes. Using 2020 United States National Health and Wellness Survey (NHWS) data, we conducted a retrospective, cross-sectional analysis to determine the associations between insomnia severity and a number of health outcomes germane to patients (health-related quality of life (HRQoL), employers and government (workplace productivity), and healthcare payers (healthcare resource utilization (HCRU)). The Insomnia Severity Index (ISI) questionnaire was used to evaluate overall insomnia severity. HRQoL was assessed using the physical and mental component summary scores of the Short Form-36v2 (SF-36v2) questionnaire, and health utility status was measured using the Short Form-6D (SF-6D) and EuroQoL-5D (EQ-5D) questionnaires. Workplace productivity was measured using the Work Productivity and Activity Impairment (WPAI) questionnaire. After adjusting for confounders, greater insomnia severity was significantly associated with worsened quality of life, decreased productivity, and increased HCRU in an apparent linear fashion. These findings have important implications for future research, including the need for specific assessment of insomnia symptoms and their impact on key health outcomes.

## 1. Introduction

Insomnia, defined as difficulty initiating and/or maintaining sleep with associated daytime consequence, is the most common sleep disorder among adults. In the United States (US), 10–15% of the population experiences chronic insomnia disorder, defined as frequency of difficulty sleeping of three or more nights per week, and duration for three or more months, with associated daytime insomnia symptoms [1,2,3]. Chronic insomnia is associated with a broad range of adverse consequences, including increased rates of poorer mental health outcomes (e.g., depression, anxiety, chronic pain, substance abuse, suicide), poorer physical health outcomes (e.g., cardiovascular disease, diabetes, stroke) and worsened health-related quality of life (HRQoL) [4]. In addition to those consequences for patients and their families, insomnia is also associated with a substantial economic burden that is borne by payers, by employers, and by society. For example, untreated insomnia is associated with increased healthcare resource utilization (HCRU) and other related costs (borne by payers), as well as increased absenteeism and diminished workplace productivity costs (i.e., presenteeism; borne by employers, but also impacting a country’s economy) [5,6,7].

Daytime impairment is a core feature of insomnia disorder. Specific daytime insomnia symptoms can include fatigue, depressed mood, irritability, poor cognitive function, increased risk of accidents, and overall impaired social, vocational, educational, and behavioral functioning [4,8,9,10]. Despite the potentially debilitating impact of these daytime insomnia symptoms, insomnia research to date has primarily focused on nighttime insomnia symptoms (i.e., difficulty initiating sleep and/or difficulty maintaining sleep) when considering adverse outcomes associated with insomnia.

In terms of the economic burden of insomnia, studies have examined insomnia, and assessed its severity, via diagnostic interview in prospective clinical studies, physician-assigned diagnoses in administrative claims studies, validated self-report instruments in survey research, and other approaches. [5,11,12,13] To our knowledge, there are limited analyses with data from a large number of patients that could be correlated with longer-term health and lifestyle outcomes. Given that insomnia is widely recognized as a 24-h disorder that impacts sleep quantity and quality and affects daytime functioning, data relating the severity of insomnia to outcomes would provide clinicians and researchers evidence-based guidance regarding the importance of insomnia assessment and the need for effective insomnia care.

To address this important gap in knowledge, the purpose of the present study was to assess the association between insomnia severity and key health outcomes that matter to diverse stakeholders, including patients (HRQoL), payers (HCRU), and employers and government (workplace productivity). For each of these outcome domains, we hypothesized that increasing insomnia severity is associated with worse health outcomes.

## 2. Materials and Methods

### 2.1. *Study Design and Data Source*

This was a retrospective, cross-sectional, observational cohort study. Data were derived from the 2020 US National Health and Wellness Survey (NHWS, Cerner Enviza, New York, NY, USA) [14,15]. The NHWS is an annual, self-administered, nationwide, internet-based survey of adults (*n* = ~75,000 US respondents aged ≥18 years) that collects demographic, general health, and disease-specific information and also includes measures of HRQoL, HCRU, and costs for more than 164 disease states. Respondents are recruited through a general-purpose, web-based consumer panel via channels such as opt-in e-mails, co-registration with panel partners, and e-newsletter campaigns. To ensure a representative sample of US adults, the NHWS employs a stratified random sampling procedure (including sex, race/ethnicity, and age). The data used included sociodemographic and general health characteristics, comorbidity burden, insomnia-related measures, current treatments, and EQ-5D-5L health states, among other data obtained from the survey respondents. Notably, while filling out the NHWS survey, if a person responded as having an insomnia diagnosis or as experiencing insomnia symptoms, then the questions of the Insomnia Severity Index (ISI) were posed within that survey. The NHWS protocol and survey were reviewed and determined exempt by the Pearl Institutional Review Board (Indianapolis, IN, USA; 19-KANT-204).

### 2.2. Participants

Participants were eligible for inclusion if they were ≥18 years old, residing in the US at the time of survey completion, and self-reported as having been diagnosed and/or experiencing insomnia during the past 12 months. Respondents were excluded from this analysis if they: (1) experienced symptoms, or had been diagnosed, with narcolepsy, sleep apnea, or other non-insomnia related sleep difficulties in the past 12 months; (2) experienced symptoms or self-reported a diagnosis of another serious medical condition (any type of cancer, chronic liver disease, cirrhosis, epilepsy, multiple sclerosis, muscular dystrophy, or Parkinson’s disease); or (3) were pregnant at the time of survey completion.

### 2.3. Insomnia Cohort Identification

Participants were identified based on responses to two standard assessment items within the NHWS: ‘Which of the following conditions have you experienced in the last 12 months?’ and ‘Which of your conditions have been diagnosed by a physician?’. Participants who reported experiencing insomnia in the previous 12 months, with or without receipt of a physician-assigned diagnosis of insomnia, completed the Insomnia Severity Index (ISI; see below). Insomnia severity was then determined based on responses to the validated ISI.

### 2.4. Insomnia Severity

Insomnia severity was assessed using the ISI, a well-established measure of insomnia symptoms that occur during both nighttime and daytime, albeit in broad categories [16]. The ISI is a 7-item self-report questionnaire assessing the nature, severity, and impact of insomnia [16,17,18,19]. Seven items are scored from 0 (indicating little/no insomnia) to 4 (indicating problems with insomnia), and total scores range from 0 to 28. Based on this summary score, insomnia severity is categorized as follows: severe insomnia (22–28), moderate insomnia (15–21), ‘subthreshold’ (mild) insomnia (8–14), and ‘no clinically significant’ insomnia (0–7) [16].

### 2.5. Outcomes

#### 2.5.1. Health-Related Quality of Life

To provide insight into the burden of insomnia from the patient perspective, HRQoL was assessed using the Medical Outcomes Study 36-Item Version 2 Short Form Survey Instrument (SF-36v2) (RAND, Santa Monica, CA, USA) [20]. SF-36v2 is a measure of general HRQoL that comprises 36 items that map onto 8 health domains: physical functioning, physical role limitations, bodily pain, general health, vitality, social functioning, emotional role limitations, and mental health. These individual domains are summarized in two component summary scores, the physical component summary (PCS) and mental component summary (MCS). PCS and MCS scores range from 0 to 100, each based on a population norm with a midpoint of 50, with higher scores indicating better HRQoL. Differences greater than 3.0 on the norm-based scoring algorithm were considered to be minimal clinically important differences (MCID) for scores on both scales [21].

#### 2.5.2. Health Utility Status

Health utilities were assessed using two established measures: the Short Form-6 Dimensions (SF-6D) and the EuroQoL-5 Dimensions (EQ-5D-5L). SF-6D health utility index scores were derived from responses on the SF-36v2 [22]. EuroQol-5D (EQ-5D) health utility index scores were derived from the EQ-5D-5L, a self-report measure of health for clinical and economic appraisal that is comprised of five dimensions: mobility, self-care, usual activities, pain/discomfort, and anxiety/depression [20]. Both SF-6D and EQ-5D health utility index scores range from 0.00 (a health state equivalent to death) to 1.00 (a health state equivalent to perfect health), with higher scores indicating better health status. Consistent with previous studies, differences greater than 0.04 and 0.07 were considered to be MCID for SF-6D and EQ-5D, respectively [22,23].

#### 2.5.3. Workplace Productivity

To provide insight into the burden of insomnia from the employer perspective, work productivity loss and non-work activity impairment were measured using the Work Productivity and Activity Impairment (WPAI) questionnaire, a validated 6-item instrument that includes four metrics: absenteeism (the percentage of work time missed because of one’s health during the past 7 days), presenteeism (the percentage of impairment due to one’s health experienced while at work during the past 7 days), overall work productivity loss (an overall impairment estimate that is a combination of absenteeism and presenteeism), and activity impairment (the percentage of impairment due to one’s health in daily activities during the past 7 days) [24]. All respondents provided data for activity impairment, but only respondents who were employed (full-time, part-time, or self-employed) provided data for absenteeism, presenteeism, and overall work impairment. Higher scores on these measures indicated greater impairment. The values ranged from no impact (0%) to complete (100%) for absenteeism, presenteeism, and overall work impairment.

#### 2.5.4. Healthcare Resource Utilization and Costs

To provide insight into the burden of insomnia from the payer perspective, HCRU was assessed based on the self-reported mean number of all-cause visits to a general practitioner and/or any healthcare provider, an emergency room (ER), or hospital during the past 6 months.

### 2.6. Analytic Plan

First, distributions and frequencies of all variables were assessed using descriptive statistics. Then, to test our hypothesis that insomnia severity is associated with worsened outcomes, we compared HRQoL, workplace productivity, and HCRU between the insomnia severity categories using ANOVA for continuous variables having normal distributions, the Wilcoxon Rank Sum test (Mann–Whitney U Test/Kruskal–Wallis) test for variables having non-Gaussian distributions, and the chi-squared test for categorical variables. Next, to control for potential confounders, we created a series of generalized linear models (GLMs) specifying a normal distribution and identity function for normally distributed outcomes (HRQoL and health status), and GLMs specifying a negative binomial distribution and log-link function for highly positively skewed variables (WPAI and HCRU). This approach to GLMs with normal and negative binomial distributions for these measures has been used previously [15,25,26]. All statistical analyses were performed using SPSS Version 23 (IBM, New York, NY, USA) or R version 3.6 or higher (RStudio, Boston, MA, USA). Statistical significance for all tests was set at *p* < 0.05.

## 3. Results

### 3.1. Characteristics of Study Population

The final sample included 8920 respondents who had experienced or been diagnosed with insomnia during the past 12 months. Of these individuals, 498 (5.6%) reported severe insomnia symptoms, 2132 (23.9%) reported moderately severe symptoms, 4348 (48.7%) reported mild (i.e., subthreshold) symptoms, and 1942 (21.8%) reported no clinically significant insomnia. Table 1 presents differences between insomnia groups. Twenty-seven percent (27.4%) of participants reported having been diagnosed with insomnia by a physician, and 13.7% reported currently taking medication to treat insomnia. Insomnia severity was positively associated with a mean number of comorbidities (0.53, 0.43, 0.29, and 0.25 for severe, moderately severe, mild, and no clinically significant insomnia, respectively; *p* < 0.001), as well as anxiety (55.6%, 48.4%, 31.2%, and 19.4%, respectively; *p* < 0.001), depression (58.4%, 47.0%, 31.9%, and 20.1%, respectively; *p* < 0.001), PTSD (15.3%, 9.4%, 4.8%, and 2.5%, respectively; *p* < 0.001) and pain (57.8%, 51.0%, 38.3%, and 30.0%, respectively; *p* < 0.001).

### 3.2. Association between Insomnia Severity and Outcomes

In unadjusted analyses, increasing insomnia severity was associated with poorer outcomes; and this pattern was evident across all outcomes examined (Table 2). Unadjusted outcome comparisons between severe, moderate, mild, and no clinically significant insomnia found that higher levels of insomnia severity were associated with worse scores for SF-6D, PCS, MCS, EQ-5D, absenteeism, overall work productivity, and overall activity impairment.

**Figure 1:**
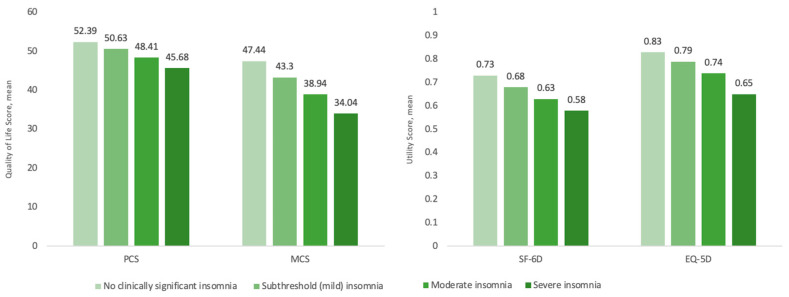
Adjusted utility values by insomnia severity category. Abbreviations: EQ-5D, EuroQol-5D; MCS, mental component summary; PCS, physical component summary; SF-6D, Short Form-6 Dimensions.

After adjusting for confounders, this linear correlation with insomnia severity persisted across all outcomes. When the insomnia cohort was stratified by severity, respondents exhibiting severe, moderate, and mild insomnia reported significantly lower EQ-5D scores (0.65 ± 0.01, 0.74 ± 0.00 and 0.79 ± 0.00, respectively) than those with no clinically significant insomnia (0.83 ± 0.00, *p* < 0.001) (Table 3). Across all other HRQoL measures, the severe, moderate, and mild groups were also associated with significantly lower SF-6D scores (0.58 ± 0.00, 0.63 ± 0.00, and 0.68 ± 0.00 vs. 0.73 ± 0.00), PCS scores (45.7 ± 0.4, 48.4 ± 0.2, and 50.6 ± 0.1 vs. 52.4 ± 0.2), and MCS scores (34.0 ± 0.5, 38.9 ± 0.2, and 43.3 ± 0.2 vs. 47.4 ± 0.2) when compared with scores for the no clinically significant insomnia group (*p* < 0.001 for all) (Figure 1, Table 3).

**Figure 2:**
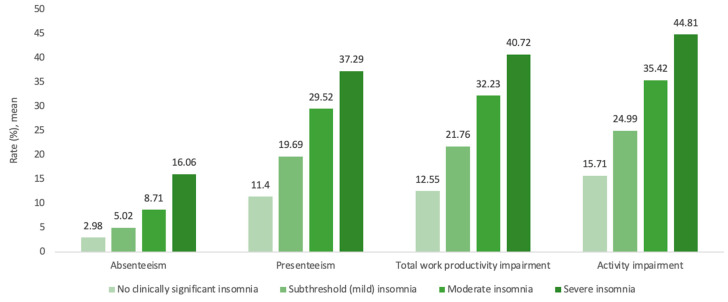
Association between work productivity and activity impairment values by insomnia severity category.

Relative to no clinically significant insomnia, severe insomnia was associated with significantly decreased work productivity, with lower scores for absenteeism (16.1 ± 3.2, rate ratio [RR] = 5.39, *p* < 0.001), presenteeism (37.3 ± 3.2, RR = 3.27, *p* < 0.001), total work productivity loss (40.7 ± 3.4, RR = 3.24, *p* < 0.001) and overall activity impairment (44.8 ± 2.3, RR = 2.85, *p* < 0.001). Proportionally similar results were also seen for moderate insomnia (absenteeism: 8.7 ± 0.9, RR = 2.92; presenteeism: 29.5 ± 1.2, RR = 2.59; total work productivity loss: 32.2 ± 1.3, RR = 2.57; overall activity impairment: 35.4 ± 0.9, RR = 2.25; all *p* < 0.001) and mild insomnia (absenteeism: 5 ± 0.3, RR = 1.68; presenteeism: 19.7 ± 0.5, RR = 1.73; total work productivity loss: 21.8 ± 0.6, RR = 1.73; overall activity impairment: 24.9 ± 0.4, RR = 1.59; all *p* < 0.001; Figure 2, Table 3).

**Figure 3:**
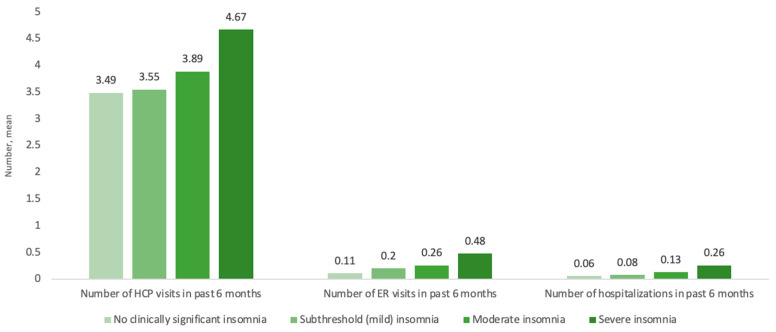
Association between HCRU by insomnia severity category. Abbreviations: ER, emergency room; HCP, healthcare professional; HCRU, healthcare resource utilization.

Increased insomnia severity was also significantly associated with higher HCRU. Specifically, respondents with severe, moderate, and mild insomnia had a higher adjusted mean number of healthcare professional visits (4.67 ± 0.27, RR = 1.34; 3.89 ± 0.11, RR = 1.11; 3.55 ± 0.07, RR = 1.02), ER visits (0.48 ± 0.06, RR = 4.19; 0.26 ± 0.02, RR = 2.31; 0.20 ± 0.01, RR = 1.73), and more hospitalizations (0.26 ± 0.04, RR = 4.26; 0.13 ± 0.01, RR = 2.11; 0.08 ± 0.01, RR = 1.34) when compared with the results for those with no clinically significant insomnia (*p* < 0.001 for all; Figure 3, Table 3).

In Supplementary Materials, a costing of the above health outcomes has been performed (Tables S3–S6), as well as an analysis of the unadjusted and adjusted associations between insomnia diagnosis status and health outcomes (Tables S1 and S2).

## 4. Discussion

In this national study, insomnia severity was associated with key outcomes that matter to patients (worsened quality of life), to payers (increased HCRU), and to employers and government (decreased work productivity and greater activity impairment). These associations highlight the importance of comprehensive clinical insomnia evaluation.

It is well-established that insomnia is associated with increased health and economic burdens, with costs borne by patients, payers, employers, and society. Our findings are, thus, consistent with and build upon previous results by utilizing the ISI, a validated measure of insomnia severity, to examine the associations between insomnia severity and a number of key outcomes that matter to patients, payers, and employers [5]. In this study, insomnia severity was linearly associated with the vast majority of health outcomes. Moreover, it is important to recognize that even the non-severe categories of insomnia were all associated with increased disease burden, highlighting not only the importance of sensitive research measures regarding insomnia severity, but also the potential clinical relevance of even mild insomnia. It is further notable that only a minority of patients who reported clinical insomnia symptoms on the ISI reported receiving a diagnosis by their physician, with even fewer reporting having treatment for their insomnia.

In aggregate, the findings of this study highlight the importance of thorough assessment of insomnia symptom severity among adult patients in real-world settings. From a harm reduction perspective, present data suggest that all patients who experience insomnia symptoms, and not just the most severe patients, should be evaluated and when indicated, considered for treatment.

Insomnia is a 24-h disorder that impacts individuals during both the night and the day. These important data are relevant to both research and clinical perspectives. From a research perspective, future studies could examine the impact of both daytime and nighttime insomnia symptom severity on other key health outcomes from varied perspectives, including the impacts on costs from the payer perspective, as well as how these treatment-related changes impact downstream health and mental health outcomes.

This study possesses multiple strengths. First, our NHWS (and ISI) data source was large and designed to represent the US adult population [27]. In addition, whereas most large-scale studies of insomnia have utilized non-validated measures, our operational definition of insomnia, the ISI, has undergone extensive psychometric validation for assessment of insomnia severity [18]. Finally, given the breadth of measures included in the NHWS, we were able to adjust for a broad range of potential confounders.

Simultaneously, our results must be interpreted in light of several important limitations. First, although NHWS is designed to mirror the US population and does control for age, sex, and self-reported race, participants are not randomly selected. It is, thus, unclear how well results will generalize to other adults. Second, all data are based on self-report, and we were unable to objectively assess sleep, HCRU, or other specific variables of interest. Third, although the ISI includes general measures of daytime insomnia symptom severity, it was neither developed nor validated to specifically assess granular domains of daytime impairment, and we were unable to assess granular daytime insomnia symptoms such as cognition, mood, or sleepiness. Fourth, although we controlled for a broad range of potential confounders, the potential for residual confounding remains. Finally, our study design was cross-sectional, and we are unable to determine causality.

## 5. Conclusions

This national analysis suggests that overall insomnia severity is strongly linearly related to adverse health outcomes including diminished HRQoL, greater activity impairment, decreased workplace productivity, and higher healthcare resource utilization. These findings add to the insomnia science literature and warrant further exploration in future studies, including prospective clinical and economic studies with long-term follow-up utilizing validated instruments.

## Figures and Tables

**Table 1:** Characteristics of study population, overall and by ISI severity group.

Categorical		Overall Population		Severe Insomnia		Moderate Insomnia		Mild (Subthreshold) Insomnia		No clinically Significant Insomnia		*p* Value
		(*n* = 8920)		(*n* = 498)		(*n* = 2132)		(*n* = 4348)		(*n* = 1942)		
		*n*	%	*n*	%	*n*	%	*N*	%	*n*	%	
Sex	Male	2776	31.1	161	32.3	570	26.7	1380	31.7	665	34.2	<0.001
	Female	6144	68.9	337	67.7	1562	73.3	2968	68.3	1277	65.8	
Marital status	Married or living with partner	4527	50.8	236	47.4	1004	47.1	2238	51.5	1049	54.0	<0.001
	Single/never married or divorced or separated or widowed	4374	49	260	52.2	1119	52.5	2102	48.3	893	46.0	
	Decline to answer	19	0.2	2	0.4	9	0.4	8	0.2	0	0.0	
Level of education	4-year university degree or higher	4132	46.3	197	39.6	818	38.4	2073	47.7	1044	53.8	<0.001
	Less than 4-year university degree	4773	53.5	301	60.4	1314	61.6	2267	52.1	891	45.9	
	Did not attend school or declined to answer	15	0.2	0	0	0	0	8	0.2	7	0.4	
Employment status	Employed full time/part time/self-employed	5301	59.4	296	59.4	1201	56.3	2655	61.1	1149	59.2	<0.001
	Not employed	2227	25	149	29.9	695	32.6	1011	23.3	372	19.2	
	Retired	1392	15.6	53	10.6	236	11.1	682	15.7	421	21.7	
Household income (categorical)	Less than $49,999	3845	43.1	263	52.8	1084	50.8	1802	41.4	696	35.8	<0.001
	$50,000 or more	4679	52.5	212	42.6	970	45.5	2353	54.1	1144	58.9	
	Decline to answer	396	4.4	23	4.6	78	3.7	193	4.4	102	5.3	
Insurance status	Yes	7774	87.2	414	83.1	1818	85.3	3807	87.6	1735	89.3	<0.001
	No	1146	12.8	84	16.9	314	14.7	541	12.4	207	10.7	
Smoking status	Current smoker	1639	18.4	157	31.5	506	23.7	720	16.6	256	13.2	<0.001
	Former smoker	2259	25.3	112	22.5	538	25.2	1131	26.0	478	24.6	
	Never	5022	56.3	229	46.0	1088	51.0	2497	57.4	1208	62.2	
Alcohol use	None	2613	29.3	162	32.5	674	31.6	1194	27.5	583	30.0	<0.001
	Low-moderate	5216	58.5	257	51.6	1207	56.6	2616	60.2	1136	58.5	
	4+ times per week	1091	12.2	79	15.9	251	11.8	538	12.4	223	11.5	
Depression		3071	34.4	291	58.4	1001	47.0	1388	31.9	391	20.1	<0.001
Post-traumatic stress disorder		532	6	76	15.3	200	9.4	207	4.8	49	2.5	<0.001
Anxiety		3040	34.1	277	55.6	1032	48.4	1355	31.2	376	19.4	<0.001
Pain		3913	43.9	288	57.8	1088	51.0	1667	38.3	582	30.0	<0.001
Insomnia, diagnosed		2445	27.4	264	53.0	858	40.2	1046	24.1	277	14.3	<0.001
Insomnia, treated		1219	13.7	139	27.9	392	18.4	511	11.8	177	9.1	<0.001
**Continuous**		**Mean**	**SD**	**Mean**	**SD**	**Mean**	**SD**	**Mean**	**SD**	**Mean**	**SD**	***p* Value**
Age (years)		45.44	16.49	41.12	14.64	42.33	15.65	45.43	16.56	49.99	16.60	<0.001
Body mass index		27.73	7.05	28.69	8.70	28.32	7.59	27.67	6.97	26.97	5.96	<0.001
Charlson comorbidity index (CCI)		0.33	0.79	0.53	0.98	0.43	0.92	0.29	0.72	0.25	0.70	<0.001
Duration of symptoms in last 12 months (days)		191.41	145.76	283.5	120.6	259.3	126.7	189.0	141.1	98.7	125.8	<0.001

Abbreviations: ISI, Insomnia Severity Index.

**Table 2:** Unadjusted outcome differences by ISI severity group.

	Severe Insomnia (*n* = 498)			Moderate Insomnia (*n* = 2132)			Mild (Subthreshold) Insomnia (*n* = 4348)			No Clinically Significant Insomnia (*n* = 1942)			*p* Value
	n	Mean	SD	n	Mean	SD	n	Mean	SD	n	Mean	SD	
SF-6D	498	0.54	0.12	2132	0.61	0.11	4348	0.68	0.11	1942	0.75	0.11	<0.001
PCS	498	43.65	12.23	2132	47.72	10.82	4348	50.89	9.05	1942	52.57	7.76	<0.001
MCS	498	30.90	12.83	2132	36.78	12.28	4348	43.43	11.29	1942	49.75	9.68	<0.001
EQ-5D	498	0.60	0.25	2132	0.72	0.16	4348	0.80	0.12	1942	0.85	0.11	<0.001
Absenteeism %	284	26.52	30.97	1140	12.28	22.41	2549	6.48	16.78	1101	3.65	13.48	<0.001
Presenteeism %	270	51.41	35.30	1124	34.86	29.51	2538	21.27	25.07	1097	11.73	20.80	<0.001
Total work productivity impairment %	267	55.88	35.87	1114	38.32	31.76	2523	23.61	27.33	1092	12.94	22.59	<0.001
Activity impairment %	498	58.27	31.29	2132	42.20	29.45	4348	26.37	26.31	1942	15.79	22.57	<0.001
Number of HCP visits in past 6 months	498	6.57	9.59	2132	4.85	6.51	4348	3.76	5.19	1942	3.27	4.46	<0.001
Number of ER visits in the past 6 months	498	0.85	1.94	2132	0.40	1.05	4348	0.24	0.74	1942	0.13	0.60	<0.001
Number of hospitalizations in the past 6 months	498	0.57	1.98	2132	0.24	1.26	4348	0.12	0.73	1942	0.09	0.74	<0.001

Abbreviations: ER, emergency room; EQ-5D, EuroQol-5D; HCP, healthcare provider; ISI, Insomnia Severity Index; MCS, mental component score; PCS, physical component score; SD, standard deviation; SF-6D, Short Form-6 Dimensions.

**Table 3:** Adjusted results showing association between outcomes by ISI severity group. Covariates adjusted for: age, sex, marital status, education, employment status, smoking status, alcohol use, body mass index, total days experienced insomnia in last 12 months, any psychological comorbidities (depression, post-traumatic stress disorder, all anxiety), all pain, CCI.

	Severe Insomnia				Moderate Insomnia				Mild (Subthreshold) Insomnia				No Clinically Significant Insomnia				*p* Value
	(*n* = 462)				(*n* = 2049)				(*n* = 4180)				(*n* = 1865)				
	Mean	SE	95% CI	B	Mean	SE	95% CI	B	Mean	SE	95% CI	B	Mean	SE	95% CI	B	
SF-6D	0.58	0.00	0.57–0.59	−0.15	0.63	0.00	0.63–0.64	−0.10	0.68	0.00	0.67–0.68	−0.05	0.73	0.00	0.72–0.73	0.00	<0.001
EQ-5D	0.65	0.01	0.64–0.66	−0.18	0.74	0.00	0.74–0.75	−0.09	0.79	0.00	0.79–0.80	−0.04	0.83	0.00	0.82–0.84	0.00	<0.001
PCS	45.68	0.39	44.92–46.44	−6.71	48.41	0.19	48.04–48.77	−3.98	50.63	0.13	50.38–50.87	−1.76	52.39	0.20	51.99–52.79	0.00	<0.001
MCS	34.04	0.47	33.13–34.96	−13.4	38.94	0.23	38.49–39.38	−8.50	43.30	0.15	43.00–43.60	−4.14	47.44	0.24	46.96–47.92	0.00	<0.001
				**RR**				**RR**				**RR**				**RR**	
Absenteeism (%)	16.06	3.23	10.83–23.81	5.39	8.71	0.86	7.18–10.57	2.92	5.02	0.32	4.43–5.69	1.68	2.98	0.30	2.44–3.64	1.00	<0.001
Presenteeism (%)	37.29	3.17	31.56–44.06	3.27	29.52	1.23	27.21–32.04	2.59	19.69	0.52	18.69–20.73	1.73	11.40	0.50	10.47–12.42	1.00	<0.001
Total work productivity impairment (%)	40.72	3.44	34.51–48.04	3.24	32.23	1.33	29.73–34.94	2.57	21.76	0.57	20.68–22.91	1.73	12.55	0.54	11.54–13.65	1.00	<0.001
Activity impairment (%)	44.81	2.30	40.51–49.56	2.85	35.42	0.89	33.72–37.20	2.25	24.99	0.42	24.18–25.83	1.59	15.71	0.42	14.90–16.56	1.00	<0.001
Number of HCP visits in past 6 months	4.67	0.27	4.16–5.24	1.34	3.89	0.11	3.67–4.12	1.11	3.55	0.07	3.41–3.69	1.02	3.49	0.11	3.27–3.72	1.00	<0.001
Number of ER visits in past 6 months	0.48	0.06	0.37–0.61	4.19	0.26	0.02	0.23–0.30	2.31	0.20	0.01	0.18–0.22	1.73	0.11	0.01	0.09–0.14	1.00	<0.001
Number of hospitalizations in past 6 months	0.26	0.04	0.20–0.35	4.26	0.13	0.01	0.11–0.16	2.11	0.08	0.01	0.07–0.10	1.34	0.06	0.01	0.05–0.08	1.00	<0.001

Abbreviations: B, beta coefficient; CI, confidence interval; EQ-5D, EuroQol-5D; ER, emergency room; HCP, healthcare provider; ISI, Insomnia Severity Index; MCS, mental component score; PCS, physical component score; SE, standard error; SF-6D, Short Form-6 Dimensions; RR, rate ratio.

## Data Availability

The NHWS is a commercial dataset. Access to the dataset used in this present paper must be requested to Cerner Enviza via the following link: https://www.cernerenviza.com/contact-us, accessed on 22 July 2020. Data access conditions will be detailed by the responsible team.

## Supplementary Materials

The following supporting information can be downloaded at: https://www.mdpi.com/article/10.3390/jcm12062438/s1, Table S1: Unadjusted outcome differences between insomnia, diagnosed and insomnia, experiencing. Table S2: Adjusted outcome differences between insomnia diagnosed and insomnia experiencing. Cost-related Supplementary Materials—*Methodology for cost calculation.* Table S3: Unit cost per visit calculation Table S4: Weekly wages estimate in US 2020, Table S5: Unadjusted results showing association between cost and insomnia severity. Table S6: Adjusted results showing association between cost and insomnia severity.
