# Supplementary material for: The Associations between Insomnia Severity and Health Outcomes in the United States
Source: J Clin Med. 2023 Mar 22;12(6):2438. doi: 10.3390/jcm12062438 (PMC10053531; doi:10.3390/jcm12062438)
Supplement: Supplementary file 1 [file jcm-12-02438-s001.zip › jcm-2255091-supplementary.pdf]

Supplementary Materials

Table S1. Unadjusted outcome differences between insomnia, diagnosed and insomnia, experiencing.

	Insomnia, Diagnosed (*n* = 2445)			Insomnia, Experiencing (*n* = 6475)			Good Sleepers (*n* = 39,262)			*p* Value
	n	Mean	SD	*n*	Mean	SD	*n*	Mean	SD	
SF–6D	2445	0.64	0.13	6475	0.68	0.12	39,262	0.75	0.14	<0.001
PCS	2445	47.30	10.72	6475	51.15	9.10	39,262	52.24	8.43	<0.001
MCS	2445	40.27	13.04	6475	43.37	12.11	39,262	48.77	10.66	<0.001
EQ–5D	2445	0.73	0.17	6475	0.80	0.15	39,262	0.87	0.15	<0.001
Absenteeism %	1278	11.47	23.47	3796	7.22	17.67	23,856	7.19	18.09	<0.001
Presenteeism %	1242	30.65	29.05	3787	21.61	27.09	23,811	17.8	28.28	<0.001
Total work productivity impairment %	1235	33.70	31.29	3761	23.84	29.24	23,694	20.41	30.69	<0.001
Activity impairment %	2445	38.89	30.12	6475	26.13	27.77	39,262	18.97	27.41	<0.001
Number of HCP visits in past 6 months	2445	5.91	7.57	6475	3.37	4.75	39,262	2.41	4.1	<0.001
Number of ER visits in past 6 months	2445	0.38	1.08	6475	0.25	0.85	39,262	0.22	1.18	<0.001
Number of hospitalizations in past 6 months	2445	0.19	0.68	6475	0.16	1.09	39,262	0.17	1.24	0.718

Abbreviations: ER, emergency room; EQ–5D, EuroQol–5D; HCP, healthcare provider; MCS, mental component score; PCS, physical component score; SD, standard deviation; SF–6D, Short Form–6 Dimensions.

Table S2. Adjusted outcome differences between insomnia diagnosed and insomnia experiencing.

	Insomnia, Diagnosed (*n* = 2365)				Insomnia, Experiencing (*n* = 6191)				*p* Value
	Mean	SE	95% Wald CI	B	Mean	SE	95% Wald CI	B	
SF–6D	0.67	0.00	0.67–0.68	0	0.67	0.00	0.67–0.68	−0.001	0.743
EQ–5D	0.78	0.00	0.77–0.78	0	0.78	0.00	0.78–0.79	0.008	0.032
PCS	49.55	0.19	49.17–49.93	0	50.47	0.11	50.25–50.68	0.918	<0.001
MCS	43.22	0.24	42.74–43.69	0	42.44	0.14	42.17–42.71	−0.773	0.009
				**RR**				**RR**	
Absenteeism (%)	6.48	0.64	5.34–7.85	1	5.70	0.30	5.15–6.32	0.881	0.283
Presenteeism (%)	21.88	0.91	20.17–23.73	1	20.33	0.45	19.47–21.22	0.929	0.137
Total work productivity impairment (%)	23.88	0.98	22.04–25.89	1	22.40	0.49	21.47–23.38	0.938	0.192
Activity impairment (%)	27.01	0.68	25.70–28.38	1	26.03	0.38	25.31–26.78	0.964	0.235
Number of HCP visits in past 6 months	4.03	0.12	3.80–4.28	1	3.54	0.06	3.42–3.66	0.878	<0.001
Number of ER visits in past 6 months	0.22	0.02	0.19–0.25	1	0.20	0.01	0.19–0.22	0.939	0.494
Number of hospitalizations in past 6 months	0.10	0.01	0.08–0.12	1	0.10	0.01	0.08–0.11	0.982	0.892

Abbreviations: B, Beta coefficient; CI, Confidence Interval; ER, emergency room; EQ–5D, EuroQol–5D; HCP, healthcare provider; ISI, Insomnia Severity Index; MCS, mental component score; PCS, physical component score; RR, rate ratio; SE, standard error; SF–6D, Short Form–6 Dimensions.

Cost-Related Supplementary Materials

Methodology for Cost Calculation

To provide further insight into the burden of insomnia from the payer perspective, direct and indirect costs were estimated using a previously validated cost analysis approach [1,2]. First, annual direct costs were calculated by imputing the average costs for the ER visits, hospitalizations, and healthcare provider visits using unit costs for each type of HCRU based on costs from the 2018 Medical Expenditure Panel Survey (MEPS) database. Next, all costs were adjusted to 2020 U.S. dollars based on the Consumer Price Index (CPI) from the Organization for Economic Co-operation and Development (OECD). Costs were then annualized. For each respondent, the number of ER visits, hospitalizations, and physician visits were multiplied by their respective unit costs to determine six-month costs for each type of HCRU; these costs were then doubled to estimate annual costs. Finally, costs of ER visits, hospitalizations, and physician visits were summed to determine total direct costs.

Annual indirect costs were calculated based on WPAI scores using the human capital method and integrating data from the 2020 Bureau of Labor Statistics (BLS) and the WPAI-GH instrument for hours missed at work and impairment at work. To dollarize these

productivity losses, median weekly wages by age and sex were obtained from the BLS, and an hourly wage was estimated. Next, for each respondent, the number of hours they missed due to absenteeism and presenteeism was multiplied by their associated hourly wage, based on their age and sex. These figures were then annualized by multiplying by 50 work weeks in a year. The annual costs for absenteeism and presenteeism are reported separately and also combined to determine total indirect costs.

Table S3. Unit cost per visit calculation.

HRU by Age Group		MEPS Unit Cost Non-Inflated (USD 2018)	Unit Cost Inflated (USD 2020)
Office-based physician visits	18–44	301	310
	45–64	343	354
	65+	279	288
Emergency room visits	18–44	1156	1191
	45–64	1202	1239
	65+	804	829
Inpatient stays	18–44	13,876	14,301
	45–64	18,523	19,090
	65+	13,495	13,908

Cost of annual doctor visits = mean expenditure per doctor visit CPI number of doctor visits in NHWS 2 Cost of annual ER visits = mean expenditure per ER visit CPI number of ER visits in NHWS 2 Cost of annual hospital visits = mean expenditure per hospital visit CPI number of hospital visits in NHWS 2 CPI, consumer price index; ER, emergency room; HRU, healthcare resource use; MEPS, medical expenditure panel survey; NHWS, national health and wellness survey; USD, United-States dollar.

Table S4. Weekly wages estimate in US 2020.

Age, Years	Median Weekly Wages in 2020	
	Males	Females
16–24	$622	$589
25–34	$952	$852
35–44	$1205	$978
45–54	$1260	$977
55–64	$1228	$955
65+	$1115	$897

Cost of absenteeism = Median weekly earnings / 40 work hours per week × hours missed work in past 7 days in NHWS × 50 work weeks per year Cost of presenteeism = Median weekly earnings/40 work hours per week × (lost productivity while at work in NHWS/10) × hours worked in past 7 days in NHWS × 50 work weeks per year. Total indirect cost = sum of cost of absenteeism and cost of presenteeism. USD, United-States.

Table S5. Unadjusted results showing association between cost and insomnia severity.

	Severe Insomnia			Moderate Insomnia			Mild (Subthreshold) Insomnia			No Clinically Significant Insomnia			*p* Value
	n	Mean	SD	*n*	Mean	SD	*n*	Mean	SD	*n*	Mean	SD	
Cost of Absenteeism	1205	8607.84	13,252.79	3774	5541.07	10,952.57	8127	2740.91	7557.81	3996	1557.33	6237.97	<0.001
Cost of Presenteeism	1205	12,187.76	13,863.82	3776	11,797.50	13,075.96	8134	8152.18	11,061.37	4002	4808.92	8998.88	<0.001
Total Indirect Costs ($)	1205	20,795.61	19,798.23	3776	17,335.63	17,769.73	8134	10,890.73	14,400.95	4002	6363.91	11,974.06	<0.001
Cost of Doctor Visits	2030	4819.57	6757.65	6873	3895.25	5256.51	14,155	2972.22	4189.54	7534	2732.53	3338.15	<0.001
Cost of ER Visits	2030	3311.68	7293.18	6873	1439.72	4386.22	14,155	754.30	3026.29	7534	426.13	2251.70	<0.001
Cost of Hospitalization Visits	2030	39,283.70	162,353.02	6873	13,004.72	62,839.89	14,155	6628.02	38,961.95	7534	5101.53	56,611.87	<0.001
Total Direct Costs ($)	2030	47,414.94	167,047.36	6873	18,339.69	66,509.57	14,155	10,354.54	41,532.04	7534	8260.19	58,022.48	<0.001
Total Costs ($)	2030	59,759.13	169,878.12	6873	27,863.82	70,063.15	14,155	16,612.77	44,574.89	7534	11,640.65	60,071.20	<0.001

Abbreviations: ER, emergency room; SD, standard deviation.

Table S6. Adjusted results showing association between cost and insomnia severity.

	Severe Insomnia				Moderate Insomnia				Mild (Subthreshold) Insomnia				No Clinically Significant Insomnia				*p* Value
	Mean	SE	CI	RR	Mean	SE	CI	RR	Mean	SE	CI	RR	Mean	SE	CI	RR	
Cost of Absenteeism ($)	6104.19	639.57	4971.00–7495.71	4.633	4256.29	244.53	3803.02–4763.59	3.231	2292.66	87.50	2127.41–2470.74	1.740	1317.49	73.26	1181.45–1469.19	1	<0.001
Cost of Presenteeism ($)	10,404.80	481.41	9502.76–11,392.46	2.213	10,719.15	269.14	10,204.42–11,259.84	2.280	7770.21	129.05	7521.36–8027.30	1.653	4701.13	115.56	4480.01–4933.16	1	<0.001
Total Indirect Costs ($)	16,799.20	762.64	15,369.02–18,362.47	2.764	15,213.09	376.24	14,493.27–15,968.67	2.503	10,174.38	166.50	9853.22–10,506.01	1.674	6078.45	146.61	5797.78–6372.70	1	<0.001
Cost of Doctor Visits ($)	3658.59	109.34	3450.44–3879.29	1.313	3121.61	49.85	3025.41–3220.87	1.120	2774.58	29.77	2716.85–2833.55	.996	2786.59	43.20	2703.20–2872.55	1	<0.001
Cost of ER Visits ($)	1468.10	130.54	1233.29–1747.61	3.793	851.28	40.36	775.74-934.16	2.199	558.79	17.95	524.70-595.09	1.444	387.05	17.81	353.67-423.59	1	<0.001
Cost of Hospitalization Visits ($)	12,117.63	1563.19	9410.47–15,603.56	3.478	6758.16	463.17	5908.70–7729.75	1.940	4245.36	197.45	3875.49–4650.54	1.219	3483.93	230.98	3059.40–3967.36	1	<0.001
Total Direct Costs ($)	20,032.28	1488.50	17,317.36–23,172.82	2.833	11,769.43	464.61	10,893.15–12,716.20	1.664	8181.03	219.00	7762.87–8621.72	1.157	7071.77	270.31	6561.33–7621.91	1	<0.001
Total Cost ($)	27,051.20	1596.11	24,096.99–30,367.59	2.666	18,458.63	581.33	17,353.71–19,633.91	1.819	13,054.89	278.39	12,520.49–13,612.09	1.286	10,148.45	309.52	9559.58–10,773.59	1	<0.001

Abbreviations: CI, Confidence Interval; ER, emergency room; RR, rate ratio; SE, standard error.

References

1. Walters, S.J.; Brazier, J.E. Comparison of the minimally important difference for two health state utility measures: EQ-5D and SF-6D. *Qual. Life Res.* **2005**, *14*, 1523–1532. https://doi.org/10.1007/s11136-004-7713-0.
2. DiBonaventura, M.D.; Gupta, S.; McDonald, M.; Sadosky, A. Evaluating the health and economic impact of osteoarthritis pain in the workforce: Results from the National Health and Wellness Survey. *BMC Musculoskelet. Disord*. **2011**, *12*, 83. https://doi.org/10.1186/1471-2474-12-83. PMID: 21527024; PMCID: PMC3110556.
